# Thyroid Cancer Knowledge and Awareness in Saudi Arabia: A Cross-Sectional Study

**DOI:** 10.7759/cureus.47888

**Published:** 2023-10-28

**Authors:** Naeem F Qusty, Alaa Jameel A Albarakati, Manal Almasary, Seham Alsalamah, Lama Alharbi, Amnah Alharthi, Ibrahim N Al Sulaiman, Tourki A. S. Baokbah, Medhat Taha

**Affiliations:** 1 Laboratory Medicine, Faculty of Applied Medical Sciences, Umm Al-Qura University, Makkah, SAU; 2 Surgery, College of Medicine, Umm Al-Qura University, Al-Qunfudah, SAU; 3 Medicine, Faculty of Medicine, Umm Al-Qura University, Al-Qunfudah, SAU; 4 Medicine, College of Medicine, King Saud Bin Abdulaziz University for Health Sciences, Riyadh, SAU; 5 Medicine, Faculty of Medicine, King Abdulaziz University, Jeddah, SAU; 6 Medicine, College of Medicine, King Khalid University, Abha, SAU; 7 Medicine, College of Medicine, Najran University, Najran, SAU; 8 Medical Emergency Services, College of Health Sciences, Umm Al-Qura University, Al-Qunfudah, SAU; 9 Anatomy, College of Medicine, Umm Al-Qura University, Al-Qunfudah, SAU

**Keywords:** participant, knowledge, saudi arabi, thyroid cancer, awarness

## Abstract

Background

Thyroid cancer incidence has been increasing worldwide over the last few decades. It is the most common endocrine cancer and is most common among females. The study contributes to filling the knowledge gap among Saudi people regarding thyroid cancer.

Objectives

This research aims to investigate the level of thyroid cancer knowledge and awareness in Saudi Arabia, identify potential knowledge gaps, and develop targeted strategies for enhancing public awareness and education.

Methods

A cross-sectional, voluntary online survey was conducted from 1st August 2023 to 1st October 2023 among residents living in Saudi Arabia over 18 years of age. The participants included were 2030 respondents. Data analysis was performed using RStudio (R version 4.3.0; R Foundation for Statistical Computing, Vienna, Austria).

Results

Among the participants, the majority were female (60.4%). A total of 49.7% of the individuals reported having a moderate to high level of knowledge about thyroid cancer. While 63.9% knew the association of a lump in the neck to thyroid cancer, 82.6% affirmed to consult a doctor upon discovering a lump, 72.1% knew that regular monitoring of neck lumps is crucial for early diagnosis and treatment of precancerous conditions, 38.7% were aware of females being prone to thyroid cancer, and 59.2% were aware of the link between lifestyle and increased risk. Higher awareness scores were positively associated with female gender, previously having thyroid function tests done, and previously undergoing a US scan of the thyroid.

Conclusion

In this study, Saudi individuals are reported to lack some aspects of knowledge and perception of thyroid cancer. This study emphasizes filling the existing knowledge gap in thyroid cancer awareness in the Saudi population.

## Introduction

The most common endocrine cancer is thyroid cancer (TC), and this frequency has dramatically grown over the last few decades [1,2]. Some medical experts think that early diagnosis utilizing cutting-edge technology is a significant influence, even though the causes of this growth are not entirely known [3-5]. Several researchers have hypothesized that this growth is the result of alterations in the environment and people's lifestyles [1,2,6]. The Global Cancer Observatory (GLOBOCAN) estimates that there are 586,000 instances of TC worldwide, with rates three times higher in women than in men [7]. In Saudi Arabia, there were 2833 cases of TC in 2020, making it the second most common cancer in women (compared to 6.2% in men) [8]. With a female-to-male ratio of 1:0.3, TC is less common in males than in women and accounts for 8.8% of all cancer cases and 12% of cases in women [9]. Although the cause of this rise in female occurrence is unknown, thyroid cells' exposure to endogenous estrogen hormone is probably a major factor [10].

The thyroid gland is a large endocrine gland with two connected lobes weighing between 20 and 30 g in adults. Furthermore, the prevalence of thyroid lesions in the gland varies between 4% and 7%, and the majority of them show no symptoms while maintaining normal thyroid hormone production [11]. More than 90% of TC patients fall into one of the three main histological subtypes: developed (papillary and follicular), poorly differentiated and anaplastic, and medullary, which arises from C cells [12].

Lack of awareness about TC and its causes, as well as other risk factors such as genetics, family history, food, radiation exposure, and environmental variables, may be a factor in the rising number of TC cases [13]. According to one study, university students in Pakistan need to learn about the risk factors for TC [13]. TC is the second most common cancer among women in Saudi Arabia, according to the Saudi Cancer Registry [14]. Evaluating the degree of TC awareness among women worldwide is essential. Unfortunately, additional data on this subject has to be published. Therefore, this study aims to evaluate the knowledge and awareness of TC among the general population in Saudi Arabia.

## Materials and methods

Study design and participants

This cross-sectional study was undertaken to evaluate the levels of knowledge and awareness regarding thyroid cancer within the Saudi population. For this purpose, an anonymous questionnaire was meticulously designed and subsequently distributed among a representative sample of the populace from 1st August 2023 to 1st October 2023. The study's focus was on Saudi residents aged 18 and above, with inclusion criteria encompassing individuals of both genders within this age group. Conversely, those who did not meet this criterion, specifically Saudi Arabian residents under the age of 18, were excluded from the study. The sample was calculated using the Raosoft Sample Size Calculator (Seattle, WA, USA). The required sample from a population of 100,000 Saudi people was found to be 383 using a 95% margin of error. The participants were recruited using a non-probability sampling technique.

Data collection

A pre-designed questionnaire was utilized with the aid of a previous research project conducted in Makkah city. A self-reported online questionnaire was used to gather data; it was distributed in Arabic and was shared via various social media platforms (Twitter and WhatsApp), friends, family members and colleagues to assist with the distribution of the survey. The questionnaire encompassed several key sections, starting with a consent form to ensure ethical participation. Subsequently, participants provided sociodemographic information, followed by sections dedicated to their practices for detecting thyroid cancer, their general perception and awareness of TC, their knowledge of TC risk factors, and finally, their awareness of the diagnosis and treatment options available for thyroid cancer.

Methods

Participants’ knowledge regarding thyroid cancer was assessed based on their responses to 16 items. Each correct answer was assigned one and incorrect responses were assigned zero. An overall knowledge score (range 0 to 16) was computed by summing the correct answers, and a percent score (range 0 to 100) was calculated to facilitate the interpretation of scores. Knowledge levels were categorized as low knowledge (0 to 50), moderate knowledge (51 to 75) and high knowledge (> 75).

Statistical analysis

**Figure 1:**
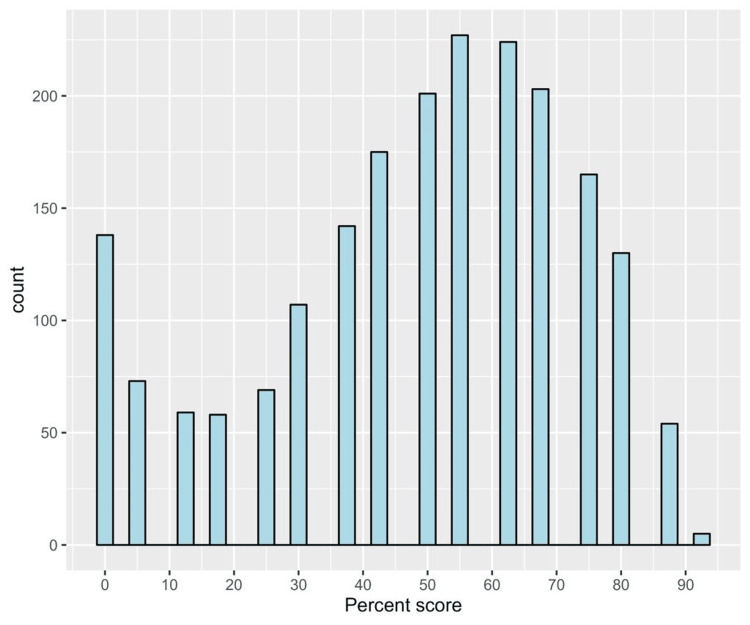
A histogram depicting the frequency distribution of the knowledge score

Data analysis was performed using RStudio (R version 4.3.0; R Foundation for Statistical Computing, Vienna, Austria). The normality of the knowledge score was assessed using a Shapiro-Wilk test, which was statistically significant (p < 0.001), indicating non-normal distribution. This was confirmed by a visual depiction of the relevant histogram (Figure 1). Therefore, we applied non-parametric testing. We expressed categorical data as frequencies and percentages, whereas numerical data were presented as median and interquartile range (IQR). The differences in knowledge scores between study groups were analyzed using a Wilcoxon rank sum test for variables with two categories or a Kruskal-Wallis rank sum test for variables with three or more categories. We applied a multivariable generalized linear regression analysis to assess the independent predictors of knowledge regarding thyroid cancer using the significantly associated variables as independent variables, and the percent knowledge score as a dependent variable. Results were presented as beta coefficients and 95% confidence intervals (95% CIs). Statistical significance was set at p<0.05. 

## Results

Sociodemographic characteristics and participants’ practices for detecting thyroid cancer

We initially received 2045 on the online platform. However, 15 records of participants aged <18 years were excluded. Therefore, data of 2030 respondents were analyzed in the current study. The majority of participants were Saudi nationals (94.8%), with females constituting a larger proportion (60.4%) than males (39.6%). The age distribution revealed that most participants fell within the 18 to 24 age group (37.9%), followed by the 25 to 44 age group (34.6%). A significant portion of the participants were married (52.0%), and the majority resided in the Eastern region of Saudi Arabia (46.5%). In terms of educational attainment, the majority held a university degree (71.9%), while the most common occupational status was "employed - non-medical" (29.4%). A substantial proportion reported a monthly income between SAR 10,000 and 20,000 (40.5%). Interestingly, a significant proportion (76.0%) indicated having a family member or friend working in healthcare. Regarding healthcare utilization, 40.5% reported visiting a health center three times or more per year. Additionally, 38.8% reported having undergone a thyroid hormone analysis, while a smaller proportion (10.4%) had received an ultrasound or CT scan of the thyroid gland (Table 1).

**Table 1:** Sociodemographic characteristics and participants’ practices for detecting thyroid cancer

Characteristic	N (%)
Nationality	
Saudi	1,925 (94.8%)
Non-Saudi	105 (5.2%)
Gender	
Male	804 (39.6%)
Female	1,226 (60.4%)
Age (year)	
18 to 24	770 (37.9%)
25 to 44	702 (34.6%)
45 or more	558 (27.5%)
Marital status	
Single	902 (44.4%)
Married	1,055 (52.0%)
Divorced	44 (2.2%)
Widowed	29 (1.4%)
Region of residence	
Eastern	944 (46.5%)
Western	550 (27.1%)
Northern	167 (8.2%)
Southern	122 (6.0%)
Central	247 (12.2%)
Educational level	
None	4 (0.2%)
Secondary school or less	406 (20.0%)
University degree	1,459 (71.9%)
Master/PhD	161 (7.9%)
Occupational status	
Student - Medical	452 (22.3%)
Student - Non-medical	206 (10.1%)
Employed - Medical	192 (9.5%)
Employed - Non-medical	597 (29.4%)
Private work	237 (11.7%)
Housewife	346 (17.0%)
Monthly income (SAR)	
<5,000	303 (14.9%)
5,000 to 10,000	445 (21.9%)
>10,000 to 20,000	823 (40.5%)
>20,000	459 (22.6%)
Having a family member or a friend working in healthcare	1,542 (76.0%)
Frequency of visits to a health center per year	
None	513 (25.3%)
Once	294 (14.5%)
Twice	401 (19.8%)
Three times or more	822 (40.5%)
Ever done a thyroid hormone analysis	787 (38.8%)
Ever undergone an ultrasound or CT scan of the thyroid gland	212 (10.4%)

Participants’ responses to knowledge items

In general, 46.0% recognized that thyroid cancer is not incurable, while 75.5% correctly indicated that the condition is not contagious. Additionally, only 6.7% were aware that thyroid cancer can be prevented, and 34.9% recognized that the disease is more common in individuals over 40 years old. About one-quarter of participants correctly stated that thyroid cancer is not uncommon in Saudi Arabia (28.9%). In terms of awareness campaigns, 75.5% had attended or watched special campaigns for thyroid cancer.

Moving to risk factors, 28.7% correctly acknowledged that thyroid cancer can be genetic, and 59.2% were aware of the association between lifestyle and increased risk. Notably, 39.5% accurately understood that the presence of a risk factor does not guarantee the disease's occurrence. Awareness of preventive measures included 58.7% recognizing that physical activity reduces risk, and 54.2% being aware that obesity increases risk.

Regarding diagnosis and treatment, 63.9% correctly identified the appearance of a lump or knot in the neck as a sign of thyroid cancer, and 72.1% knew that monitoring neck swelling aids early detection. Impressively, 82.6% affirmed they would consult a doctor upon discovering a lump or knot. Finally, 7.2% correctly identified males and 38.7% identified females as more prone to thyroid cancer. An overall low proportion (10.1%) had attended awareness campaigns (Table 2).

**Table 2:** Participants' responses to knowledge items. *An asterisk indicates a correct response

Characteristic	N (%)
General perception and awareness of thyroid cancer	
Thyroid cancer is incurable	
No*	933 (46.0%)
Yes	315 (15.5%)
Do not know	782 (38.5%)
Thyroid cancer is contagious	
No*	1,533 (75.5%)
Yes	66 (3.3%)
Do not know	431 (21.2%)
Thyroid cancer can be prevented	
No*	135 (6.7%)
Yes	1,294 (63.7%)
Do not know	601 (29.6%)
Thyroid cancer is uncommon in Saudi Arabia	
No*	587 (28.9%)
Yes	365 (18.0%)
Do not know	1,078 (53.1%)
Thyroid cancer is more common in those who are older than 40 years	
No	221 (10.9%)
Yes*	708 (34.9%)
Do not know	1,101 (54.2%)
Ever attended or watched the effectiveness or special awareness campaign for thyroid cancer	
No	66 (3.3%)
Yes*	1,533 (75.5%)
Do not know	431 (21.2%)
Awareness of the risk factors of thyroid cancer	
Thyroid cancer is often genetic	
No	455 (22.4%)
Yes*	582 (28.7%)
Do not know	993 (48.9%)
Lifestyle is associated with an increased risk of thyroid cancer, for example, stability or diet	
No	164 (8.1%)
Yes*	1,201 (59.2%)
Do not know	665 (32.8%)
The presence of a risk factor for thyroid cancer means that the disease that I I’d be	
No*	802 (39.5%)
Yes	402 (19.8%)
Do not know	826 (40.7%)
Physical activity reduces the risk of thyroid cancer	
No	143 (7.0%)
Yes*	1,191 (58.7%)
Do not know	696 (34.3%)
Obesity increases the risk of thyroid cancer	
No	162 (8.0%)
Yes*	1,100 (54.2%)
Do not know	768 (37.8%)
Awareness of the diagnosis and treatment of thyroid cancer	
Thyroid cancer appears in the form of a lump or knot in the neck	
No	87 (4.3%)
Yes*	1,297 (63.9%)
Do not know	646 (31.8%)
Monitoring the presence of swelling in the neck is useful for the early detection of thyroid cancer	
No	96 (4.7%)
Yes*	1,463 (72.1%)
Do not know	471 (23.2%)
If you find a lump or knot in the thyroid area, you will visit the doctor for a consultation	
No	49 (2.4%)
Yes*	1,677 (82.6%)
Do not know	304 (15.0%)
Thyroid cancer is more common in (males/females)	
Males*	146 (7.2%)
Females*	785 (38.7%)
Do not know	1,099 (54.1%)
Ever attended or watched the effectiveness or special awareness campaign for thyroid cancer	
No	1,825 (89.9%)
Yes*	205 (10.1%)

Description of the knowledge score

**Figure 2:**
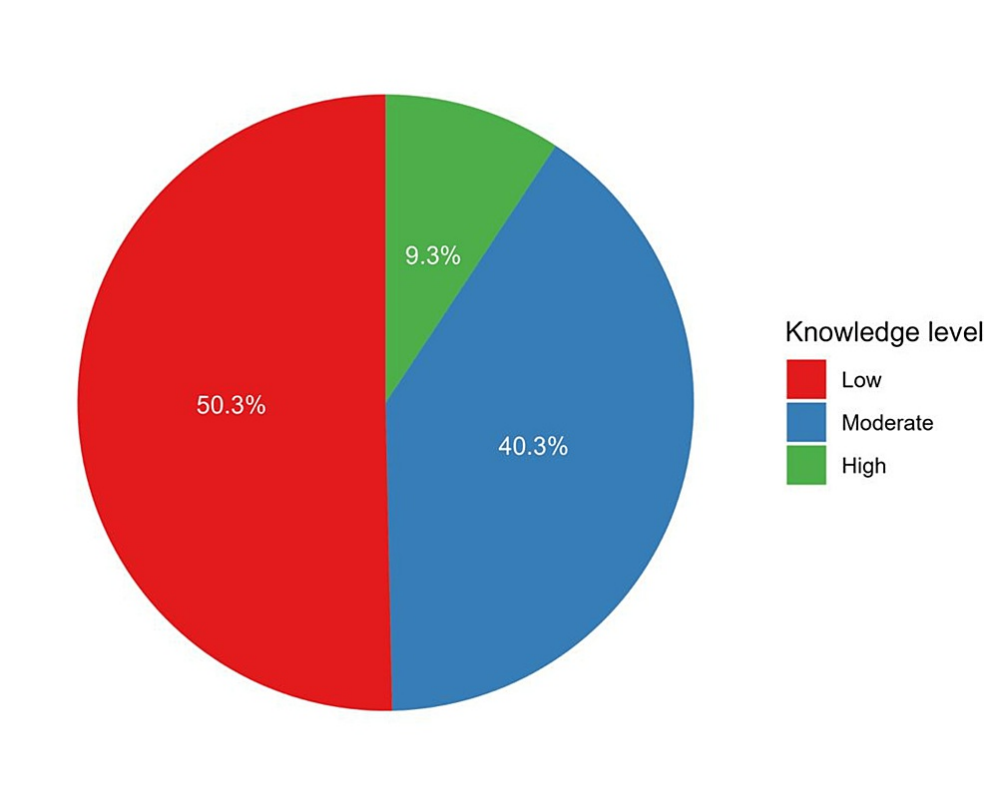
A pie chart depicting the proportions of knowledge levels

Knowledge items showed a very good level of internal consistency as indicated by a Cronbach’s alpha value of 0.834. The median knowledge score was 50.0 (IQR = 37.5 to 68.8). The distribution of knowledge scores among participants is shown in Figure 1. Approximately half of the respondents had a low level of knowledge (50.3%), while 40.3% and 9.3% of them had moderate and high levels of knowledge, respectively (Figure 2).

Differences in knowledge scores based on sociodemographic characteristics and participants’ practices for detecting thyroid cancer

Significant differences in knowledge scores based on various sociodemographic characteristics and participants' practices for detecting thyroid cancer were observed. Notably, gender exhibited a statistically significant difference (median = 50.0, IQR = 25.0 to 62.5 among males and median = 56.3, IQR = 37.5 to 68.8 among females, p<0.001). Nationality also displayed a significant difference in knowledge scores (median = 50.0, IQR = 31.3 to 68.8 among Saudis and median = 56.3, IQR = 37.5 to 68.8 among non-Saudis, p=0.047). Age groups also displayed significant discrepancies, with the 18 to 24 age group showing higher knowledge scores (median = 56.3, IQR = 37.5 to 68.8) compared to the other age groups (25 to 44: median = 50.0, IQR = 31.3 to 62.5; 45 or more: median = 50.0, IQR = 31.3 to 62.5, p<0.001). Marital status revealed statistically significant differences as well, where singles exhibited higher knowledge scores (median = 56.3, IQR = 37.5 to 68.8), as did participants from the Western (median = 56.3, IQR = 37.5 to 68.8) and Southern (median = 56.3, IQR = 32.8 to 68.8) regions, and those with a university degree (median = 56.3, IQR = 37.5 to 68.8, p<0.05 for all). Furthermore, occupational status demonstrated significant disparities, with participants in medical-related occupations showing higher knowledge scores (e.g., Student - Medical: median = 62.5, IQR = 56.3 to 75.0; Employed - Medical: median = 62.5, IQR = 50.0 to 75.0) compared to other categories (p<0.001). Participants who had ever done a thyroid hormone analysis also exhibited greater knowledge (median = 56.3, IQR = 43.8 to 68.8) compared to those who hadn't (median = 50.0, IQR = 31.3 to 62.5, p<0.001). Lastly, those who had undergone an ultrasound or CT scan of the thyroid gland had higher knowledge scores (median = 56.3, IQR = 37.5 to 68.8) than those who hadn't (median = 50.0, IQR = 31.3 to 68.8, p=0.027) (Table 3).

**Table 3:** Differences in knowledge scores based on sociodemographic characteristics and participants’ practices for detecting thyroid cancer

Characteristic	Median (IQR)	p-value
Nationality		0.047
Saudi	50.0 (31.3, 68.8)	
Non-Saudi	56.3 (37.5, 68.8)	
Gender		<0.001
Male	50.0 (25.0, 62.5)	
Female	56.3 (37.5, 68.8)	
Age (year)		<0.001
18 to 24	56.3 (37.5, 68.8)	
25 to 44	50.0 (31.3, 62.5)	
45 or more	50.0 (31.3, 62.5)	
Marital status		<0.001
Single	56.3 (37.5, 68.8)	
Married	50.0 (31.3, 62.5)	
Divorced	50.0 (37.5, 62.5)	
Widowed	50.0 (37.5, 68.8)	
Region of residence		0.006
Eastern	50.0 (31.3, 62.5)	
Western	56.3 (37.5, 68.8)	
Northern	50.0 (37.5, 62.5)	
Southern	56.3 (32.8, 68.8)	
Central	56.3 (37.5, 71.9)	
Educational level		0.003
None	59.4 (42.2, 67.2)	
Secondary school or less	50.0 (25.0, 62.5)	
University degree	56.3 (37.5, 68.8)	
Master/PhD	56.3 (37.5, 68.8)	
Occupational status		<0.001
Student - Medical	62.5 (56.3, 75.0)	
Student - Non-medical	50.0 (31.3, 56.3)	
Employed - Medical	62.5 (50.0, 75.0)	
Employed - Non-medical	43.8 (31.3, 62.5)	
Private work	43.8 (18.8, 62.5)	
Housewife	50.0 (31.3, 62.5)	
Monthly income (SAR)		<0.001
<5,000	56.3 (37.5, 68.8)	
5,000 to 10,000	50.0 (31.3, 62.5)	
>10,000 to 20,000	50.0 (37.5, 68.8)	
>20,000	56.3 (37.5, 68.8)	
Having a family member or a friend working in healthcare		0.155
No	50.0 (31.3, 68.8)	
Yes	56.3 (37.5, 68.8)	
Frequency of visits to a health center per year		0.248
None	56.3 (37.5, 68.8)	
Once	50.0 (32.8, 68.8)	
Twice	56.3 (31.3, 68.8)	
Three times or more	50.0 (31.3, 68.8)	
Ever done a thyroid hormone analysis		<0.001
No	50.0 (31.3, 62.5)	
Yes	56.3 (43.8, 68.8)	
Ever undergone an ultrasound or CT scan of the thyroid gland		0.027
No	50.0 (31.3, 68.8)	
Yes	56.3 (37.5, 68.8)	

Predictors of high knowledge scores

Based on the multivariable regression analysis, gender significantly influenced knowledge scores, with females displaying a positive association (beta = 5.97, 95% CI = 3.75 to 8.19, p<0.001), while age categories, marital statuses, and regions of residence did not exhibit significant associations. Among educational levels, participants with secondary school education or less, a university degree, or a Master's/PhD showed no significant differences in knowledge scores compared to those with no education. Occupational status had a pronounced impact on knowledge, as participants in non-medical student roles (beta = -16.8, 95% CI = -20.4 to -13.1, p<0.001), employed non-medical positions (beta = -16.6, 95% CI = -20.4 to -12.7, p<0.001), private work (beta = -17.7, 95% CI = -21.9 to -13.4, p<0.001), and housewives (beta = -18.3, 95% CI = -22.5 to -14.2, p<0.001) exhibited significantly lower knowledge scores. Participants with a monthly income of SAR 5,000 to 10,000 (beta = -4.68, 95% CI = -7.97 to -1.39, p=0.005) and SAR >10,000 to 20,000 (beta = -3.70, 95% CI = -6.80 to -0.61, p=0.019) also showed lower knowledge scores compared to those earning less than SAR 5,000. Conversely, having ever done a thyroid hormone analysis (beta = 5.73, 95% CI = 3.47 to 8.00, p<0.001) was positively associated with higher knowledge scores (Table 4).

**Table 4:** Predictors of high knowledge scores

Characteristic	Beta	95% CI	p-value
Nationality			
Saudi	Reference	Reference	
Non-Saudi	-0.15	-4.61, 4.30	0.946
Gender			
Male	Reference	Reference	
Female	5.97	3.75, 8.19	<0.001
Age (year)			
18 to 24	Reference	Reference	
25 to 44	-2.63	-6.01, 0.75	0.127
45 or more	-2.64	-6.63, 1.36	0.196
Marital status			
Single	Reference	Reference	
Married	1.70	-1.46, 4.85	0.292
Divorced	-0.27	-7.35, 6.80	0.940
Widowed	2.75	-5.96, 11.5	0.536
Region of residence			
Eastern	Reference	Reference	
Western	0.60	-1.85, 3.05	0.632
Northern	-3.06	-6.73, 0.62	0.103
Southern	-1.05	-5.27, 3.16	0.624
Central	0.04	-3.24, 3.32	0.982
Educational level			
None	Reference	Reference	
Secondary school or less	-9.85	-31.7, 12.0	0.377
University degree	-6.98	-28.8, 14.8	0.530
Master/PhD	-3.44	-25.5, 18.6	0.760
Occupational status			
Student - Medical	Reference	Reference	
Student - Non-medical	-16.8	-20.4, -13.1	<0.001
Employed - Medical	-1.56	-5.92, 2.80	0.484
Employed - Non-medical	-16.6	-20.4, -12.7	<0.001
Private work	-17.7	-21.9, -13.4	<0.001
Housewife	-18.3	-22.5, -14.2	<0.001
Monthly income (SAR)			
<5,000	Reference	Reference	
5,000 to 10,000	-4.68	-7.97, -1.39	0.005
>10,000 to 20,000	-3.70	-6.80, -0.61	0.019
>20,000	-2.40	-5.82, 1.03	0.170
Ever done a thyroid hormone analysis			
No	Reference	Reference	
Yes	5.73	3.47, 8.00	<0.001
Ever undergone an ultrasound or CT scan of the thyroid gland			
No	Reference	Reference	
Yes	2.69	-0.68, 6.06	0.118

## Discussion

Individuals have been paying more attention to metabolic diseases due to the rising incidence in recent years [15]. Among these conditions, thyroid diseases are among the best known. The prevalence of thyroid diseases is on the rise in both Saudi Arabia and the international world. However, there is a scarcity of literature available on the knowledge of individuals about thyroid disease, nationally and internationally [16-19]. Early detection of nodules and abnormal thyroid conditions can lead to faster care and lower morbidity and mortality rates [20]. This study will help interventionists and public health analysts to reduce the risk of predisposing factors and help in the development of strategies to improve the level of awareness and education for thyroid cancer.

In this study, 40.3% of the individuals reported having a moderate level of knowledge. A similar study in 2019 reported that 56% of Saudi individuals have a moderate knowledge of thyroid [21]. Moreover, in 2021, AL Qahtani (2021) reported moderate knowledge of 41.5% of the thyroid [17]. On the contrary, a study in 2021 reported that 43.9% of individuals with good knowledge [22]. Moreover, a study conducted by Almuzaini et al. (2019) showed that 57.32% of adults in Saudi Arabia have a good knowledge of the thyroid [21]. However, Alyahya et al. (2021) conducted a study in 2021 to assess the general knowledge about thyroid disease, reporting that 14.2% of the Saudis in the eastern province are knowledgeable [23]. Therefore, the data shows the prevalence of poor to moderate knowledge nationally and internationally.

Thyroid diseases are prevalent, and the symptoms appear mildly and slowly. This might be the reason for the decreased awareness trend [19,21,23]. In both males and females, thyroid cancer can lead to worse outcomes [22,24,25].

Our study shows that the knowledge score is different among genders, with females being more aware. This shows gender as a statistically significant factor (p<0.001). Another study showed that knowledge scores were significantly different concerning genders, with 60.3% as females [26]. Another study similarly reported to have a knowledge ratio of 2.2:1 female to male [21].

Our study showed that 63.9% of individuals identified the early sign of thyroid cancer as a lump or knot in the neck. Similarly, in Abdulrahman (2018) study, neck swelling was reported as a symptom of thyroid cancer [19]. Another study by Alyahya et al. (2021) identified neck swelling as a sign of thyroid cancer known to 70.6% of people [23].

In terms of risk factors, 38.7% of Saudis correctly identified that female is more prone to thyroid cancer. Similarly, another study conducted recently reported that female gender and smoking are significant risk factors for thyroid diseases [23]. AL Qahtani (2021) reported that 58.4% of people knew that being female increased the risk of thyroid diseases [17]. A study conducted in 2019 reported 33.6% female gender identification in connection to thyroid diseases [21,13,27,28]. According to the Saudi Cancer Registry, thyroid cancer is the second most common cancer among women in Saudi Arabia. Therefore, the evaluation of the awareness level of thyroid cancer is essential, especially among women.

Our study showed that only 28.7% of people correctly knew the role of genetics in thyroid cancer. In contrast, a study among Saudi females reported that 68.5% of participants were aware of the genetic role in thyroid cancer [10]. The study by AL Qahtani (2021) reported the knowledge of 58.4% of people of genetics contribution [17]. Furthermore, the American Cancer Society and Endocrine Web reported that family history, sex, age, and radiation exposure were the vital risk factors for thyroid cancer [29].

Consistent with our study, another study showed the relation of obesity as a risk factor for thyroid cancer [30-32]. As per our study, more than half (54.2%) were aware that obesity increases the risk.

A significant difference was observed in knowledge scores of participants based on their age and gender. Younger people aged 18 to 24 showed higher knowledge levels than other age groups, and most of the females statistically showed a knowledge difference [10]. Similar results were observed in a study conducted in 2023 [33,34]. In contrast to that, a study conducted in India showed that most women participants had inadequate knowledge about thyroid disorders. Regarding marital status, we found that single people had more awareness regarding thyroid cancer than married people, which is consistent with the study in Asir [10].

In our study, 46% of people correctly recognized that thyroid cancer is curable, while 75.5% were correct in saying that this is not contagious. Similarly, in the Asir region study, the belief in no cure was only among 14.9% of individuals [10]. In contrast, a study conducted among women in Makkah regarding thyroid cancer reported that 56% of ladies believed it to be incurable [14]. Advanced treatment options, research studies, and public awareness can reduce the mortality rates of thyroid cancer.

In terms of awareness campaigns, only 10.1% had attended some special thyroid cancer campaign or watched it. Most of the participants of research on thyroid cancer awareness in Makkah reported a similarly low rate [14]. This emphasizes the need for more effective campaigns and special awareness programs against thyroid cancer.

Limitations

A limitation of our study is that it is an online survey, and the illiterate might have limited this study. Secondly, it is a cross-sectional study, so we can’t explore the cause-effect associations. Thirdly, it is limited to the people of Saudi Arabia, where most participants were from Western and Southern regions. Moreover, the methodology used was a convenience sampling technique that can affect the generalization. The survey was anonymous and entirely voluntary, so the clinical data have been accurately captured. The sample size was quite large, making the results quite strong.

## Conclusions

Thyroid cancer prevalence has been increasing, as studied in research nationally and internationally. Our findings indicate that Saudi individuals lack some aspects of knowledge of thyroid cancer. The lack of awareness can have negative effects in the form of negligence, delay in consultation, and misconceptions. Moreover, 89.9% have never attended awareness campaigns for thyroid cancer. These statistics emphasize the need to address the barriers to awareness and increase the sources of information on social media, public, and healthcare platforms. Targeted strategies should be made to address this issue of potential knowledge gaps.
